# Opioid-free anesthesia—caution for a one-size-fits-all approach

**DOI:** 10.1186/s13741-020-00147-3

**Published:** 2020-06-18

**Authors:** Sushan Gupta, Avani Mohta, Vijaya Gottumukkala

**Affiliations:** Department of Anesthesiology & Perioperative Medicine, The University of Texas MD Anderson Cancer Center, Houston, Texas USA

**Keywords:** Opioids, Postoperative pain, Opioid free, Opioid sparing, Postoperative pain, Optimal analgesia, Opioid misuse, Opioid crisis

## Abstract

Post-operative pain management should ideally be optimized to ensure patient’s mobilization and ability to partake in effective pulmonary exercises for patient’s early recovery. Opioids have traditionally been the main mode for analgesia strategy in the perioperative period. However, the recent focus on opioid crisis in the USA has generated a robust discussion on rational use of opioids in the perioperative period and also raised the concept of “opioid-free anesthesia” in certain circles. Opioid-related adverse drug events (ORADE) and questionable role of opioids in cancer progression have further deterred some anesthesiologists from the routine perioperative use of opioids including their use for breakthrough pain. However, judicious use of opioid in conjunction with the use of non-opioid analgesics and regional anesthetic techniques may allow for optimal analgesia while reducing the risks associated with the use of opioids. Importantly, the opioid epidemic and opioid-related deaths seem more related to the prescription practices of physicians and post-discharge misuse of opioids. Focus on patient and clinician education, identification of high-risk patients, and instituting effective drug disposal and take-back policies may prove useful in reducing opioid misuse.

## Introduction

Poorly managed acute pain is a major deterrent in a patient’s early recovery after surgery. The Institute of Medicine in the USA reports the problem of undertreated acute post-operative pain to be as high as 80% (Gan et al. 2014). Undermanaged acute postoperative pain impedes key milestones for early recovery and increases the risk for postoperative pulmonary complications (Shea et al. 2002; Warner 2000). Furthermore, undertreated acute postoperative pain may also be associated with persistent post-surgical pain (PPSP) in 10–50% patients (Chapman and Vierck 2017; Kehlet et al. 2006).

Clinicians have traditionally used opioids to manage moderate to severe post-operative pain either as monotherapy or lately as a part of multimodal analgesia. Intraoperatively, opioids have played a central role in balanced anesthesia as they helped with nociception control and hemodynamic optimization (Egan 2019). However, opioid-associated side effects and opioid misuse have influenced the current opinionated discussions on opioid free anesthesia (OFA). OFA is the use of regional analgesia and multiple non-opioid drugs with different mechanisms of action to provide adequate analgesia and avoid opioid consumption. OFA has mainly been a discussion topic among anesthesia providers. However, since the misuse of opioids and its impact on patient’s health extends outside the operation room, OFA should be a multi-specialty prerogative involving anesthesiologists, surgeons, and pain specialists, and its ambit should include the complete perioperative period.

Opioid-related adverse drug events (ORADE) not only lead to increased patient’s morbidity and poor perioperative experience but also add to the cost of healthcare. Opioid-induced respiratory depression (OIRD) is the most worrisome of ORADE and also the main cause of opioid-related deaths (Gupta et al. 2018). In addition, opioid-induced hyperalgesia (OIH) and the pre-clinical studies linking mu receptor activity and cancer progression in various cancer cell lines have also contributed to OFA discussions (Aich et al. 2016; Lee et al. 2017; Shah et al. 2017).

Recently, however, misuse, abuse, tolerance, addiction, and diversion of opioids have emerged as a major public health concern in the USA, reaching epidemic proportions. In the last decade, there has been a sixfold increase in the misuse of opioids, contributing to 68% of all the overdose-related deaths. It is reported that a fifth of all the patients who had misused prescription opioids were opioid naïve (US Department of Health and Human Services 2017). Furthermore, it has been found that 10% of opioid-naïve patients continue chronic opioid use 1 year after curative-intent cancer surgery (Lee et al. 2017). In fact, the associated risk of a patient becoming a chronic opioid user after initial exposure in the postoperative period increases every day starting from the third postoperative day (Shah et al. 2017). Although the enthusiasm for OFA is justified with several randomized trials providing strong evidence of equivalent postoperative analgesia with OFA when compared to opioid-based anesthesia techniques, there are still many challenges that are yet to be addressed (Frauenknecht et al. 2019).

First and foremost is the confusion behind opioid-free anesthesia’s accurate definition. While the terminology opioid-free is quite extensively used, a recent meta-analysis found 15 out of 23 studies comparing OFA and opioid-based anesthesia actually used parenteral opioids in the post-operative period (Frauenknecht et al. 2019). It therefore only seems more prudent to state that most of the current evidence supports the use of opioid-minimizing techniques. A multi-modal approach as a part of opioid-sparing technique helps to minimize the use of opioids perioperatively. However, our scarce knowledge regarding the ideal combination of non-opioid adjuncts, their appropriate doses, and individual side effects pose additional challenges that preclude us from strongly advocating for complete opioid-free anesthesia techniques despite existing enthusiasm.

The second argument against going opioid-free is based on the evidence that opioid-related adverse drug events and opioid-induced respiratory depression are dose-dependent and hence the risk can be possibly mitigated by better understanding of the pharmacology of opioids and carefully titrating the doses along with methods of opioid delivery to the desired effect in each individual patient (Gupta et al. 2018; Lee et al. 2015; Weingarten et al. 2016; Weingarten et al. 2015), as well as by minimizing or avoiding the concurrent use of other sedative agents in high-risk patients. Hence, despite this recent increase in the use of non-opioid adjuncts in acute care settings, rational use of opioids still has an important role in breakthrough pain management.

The third is conflicting data with respect to the role of opioids in cancer progression and recurrence in clinical studies (Cronin-Fenton et al. 2015; Diaz-Cambronero et al. 2018; Oh et al. 2017). Currently, there is no level 1 evidence linking perioperative use of opioids for optimal pain management with increased risk for cancer progression and recurrence.

And lastly, whether rational intra- or post-operative use of opioids during hospital stay adds to opioid addiction after discharge is debatable. In fact, a major contributory factor to the opioid epidemic has been over-prescription of opioids at the time of hospital discharge (Humphreys 2017; Volkow and McLellan 2016). And minimizing perioperative use of opioid has not truly impacted physician prescription practices. A recent study comparing opioid prescription before and after application of ERAS®-based multimodal opioid-free techniques did not find a difference in the two groups with respect to the opioid prescription post-hospital discharge of patients (Brandal et al. 2017). A significant number of patients are still being discharged with opioid prescriptions despite not being on an opioid on the last day of hospital stay post-surgery (Beloeil 2019; Chen et al. 2018; Magoon and Choudhury 2019). It is reported that only a third of prescribed opioid medications at the time of discharge are used by the patient, and frequently, the medications are left unsecured in the home environment (Bartels et al. 2016). Unused opioids in unsecured environments lead to a potential diversion of medications thus contributing to abuse potential (Bartels et al. 2016; Bicket et al. 2017). It seems, therefore, more pertinent to lay emphasis on educating physicians and all clinicians’ ensuring careful procedure and patient-specific opioid prescription guidelines during the perioperative period, along with rigorous monitoring of post-discharge opioid use, and robust and safe drug disposal and “drug take-back policy.” This comprehensive approach to address the opioid crisis is lacking in our current practice and offers an immense opportunity for improvement (Kumar et al. 2019).

While most physicians are aware of the current opioid epidemic problem, certain aspects still need to be detailed. First and foremost is the importance of adequate postoperative pain management. While it is necessary for patient’s optimal recovery, enhancing satisfaction and experience with the overall surgical journey, inadequate acute pain relief also increases the risk of chronic pain and hence begets longer use of analgesic drugs, further increasing the risk of opioid misuse post-discharge (Sebastian and Shanmuganathan 2019). Brat et al. in fact found the duration of opioid use rather than the dose to be more closely associated with opioid misuse (Brat et al. 2018). Instant-release formulations (and not slow-release preparations) should be preferred in the perioperative period especially for breakthrough pain to reduce the risk of ORADE and for safer titration of opioids for optimal pain relief in both the high-risk and opioid-naïve patients (Sebastian and Shanmuganathan 2019). In our opinion, there needs to be a rational, individualized, and patient-centered approach in managing perioperative pain needs of the patient, and while we advocate and strongly encourage the use of a perioperative multi-modal approach using regional analgesia when appropriate, the pain management plan needs to be both patient- and procedure-specific. If postoperative pain relief using opioid-sparing multimodal analgesia techniques is inadequate for a patient, opioid analgesics should be judiciously used for breakthrough pain management. Our main message, therefore, in addition to the lack of robust evidence for total “opioid-free anesthesia,” is also to highlight the importance of rational post-operative use of opioids for optimal pain experience of our patients.

And while a significant emphasis is laid on training the first responders, and patients on chronic opioid therapy in the use of naloxone for management of opioid overdose, it is also the responsibility of physicians to identify red flag signs in patient’s psychosocial and behavioral patterns (preoperative opioid use, depression, substance use disorder, preoperative pain, and tobacco use) which puts them at risk for tolerance, misuse, and abuse of opioids. Although pain management strategies and principles during hospital stay are generally monitored, it is imperative that physicians and nurses understand the importance of opioid titration and the expected need for the duration of opioid/analgesic use after discharge. Each refill and additional week of opioid prescription in the post-operative period is associated with large increase of opioid misuse in opioid-naïve patients (Brat et al. 2018). In fact, Wu et al. recommend setting up a multi-disciplinary transitional pain service to reduce the risk of opioid misuse in high-risk patients (Wu et al. 2019).

It is crucial that we fully understand the behavioral, psychosocial, and medical reasons for prescription opioids becoming a source of diversion, abuse, tolerance, addiction, and overdose deaths. Instead of uniformly focusing on an “opioid-free plan” for every surgical patient and compromise on patient’s optimized comfort and early functional recovery, we should emphasize on strict checks applicable at various timelines during a patient’s surgical journey to identify and avoid misuse of opioids.

## Conclusion

Opioid-related adverse events, opioid misuse, and the current opioid crisis have fueled the current discussion on opioid-free anesthesia. Robust data on total opioid-free anesthesia is still lacking. Judicious and individualized use of opioids especially for breakthrough pain in the postoperative pain will significantly enhance patient experience and allow for faster recovery while minimizing its side effects. Patient and clinician education, identification of high-risk patients for tolerance and addiction potential, optimal titration of doses when needed, and effective monitoring and “drug take-back” programs in the post-discharge phase of care will reduce opioid misuse and opioid-related deaths.

## Data Availability

Not applicable

## Abbreviations

PPSP: Persistent post-surgical pain
OFA: Opioid-free anesthesia
ORADE: Opioid-related adverse drug events
OIRD: Opioid-induced respiratory depression
OIH: Opioid-induced hyperalgesia
